# First report of an inherited MYCBP2 neurodevelopmental disorder: review of proband and parent presentation

**DOI:** 10.1007/s10048-025-00854-3

**Published:** 2026-01-16

**Authors:** Alice Pham, Jennifer Harmon, Lia K. Thibodaux, Laura A. Flashman, Michelle Curtin

**Affiliations:** 1 Department of Pediatrics, Wake Forest University School of Medicine, Winston-Salem, NC USA; 2 Department of Neurology, Wake Forest University School of Medicine, Winston-Salem, NC USA

**Keywords:** Neurodevelopmental disorder, Inherited, Family phenotype

## Abstract

*MYCBP2*-associated neurodevelopmental disorder is an autosomal dominant genetic disorder, previously described with de novo variants. We present the case of a two-generation review of a proband with a maternally inherited heterozygous pathogenic variant in *MYCBP2*, c.4409dup (p.Leu1470Phefs*7). Neuropsychology assessment indicated developmental delays with proband’s scores falling well below age-level expectations, while proband’s mother demonstrated generally intact cognition with evidence of subtle executive inefficiency. Assessment of parental genotype and phenotype can help to anticipate child’s developmental trajectory, especially in genetic disorders associated with highly variable expressivity. However information gaps on familial impact of inherited neurodevelopmental disorders across generations remain.

## Introduction

*MYCBP2*-associated neurodevelopmental disorder is a relatively newly described autosomal dominant genetic disorder [1]. Significant characteristics include intellectual disability, developmental delays, epilepsy, facial dysmorphisms, ocular abnormalities, corpus callosum abnormalities, and autistic features. Previously all known individuals (*N* = 8) have been described with de novo variants. Here we present the case of a proband with a maternally inherited *MYCBP2*-associated neurodevelopmental disorder. Neuropsychological assessment was obtained for both proband and mother who also had the same variant.

## Case presentation

The 4-year-old proband's past medical history is significant for prematurity at 34 weeks of gestation, global developmental delay, autism spectrum disorder (ASD), chronic constipation with at least one episode of fecal impaction, sleep disturbances, and aggressive behaviors, which included biting, scratching, and pinching. Language delay was first identified at his 15-month well child check.

**Fig. 1:**
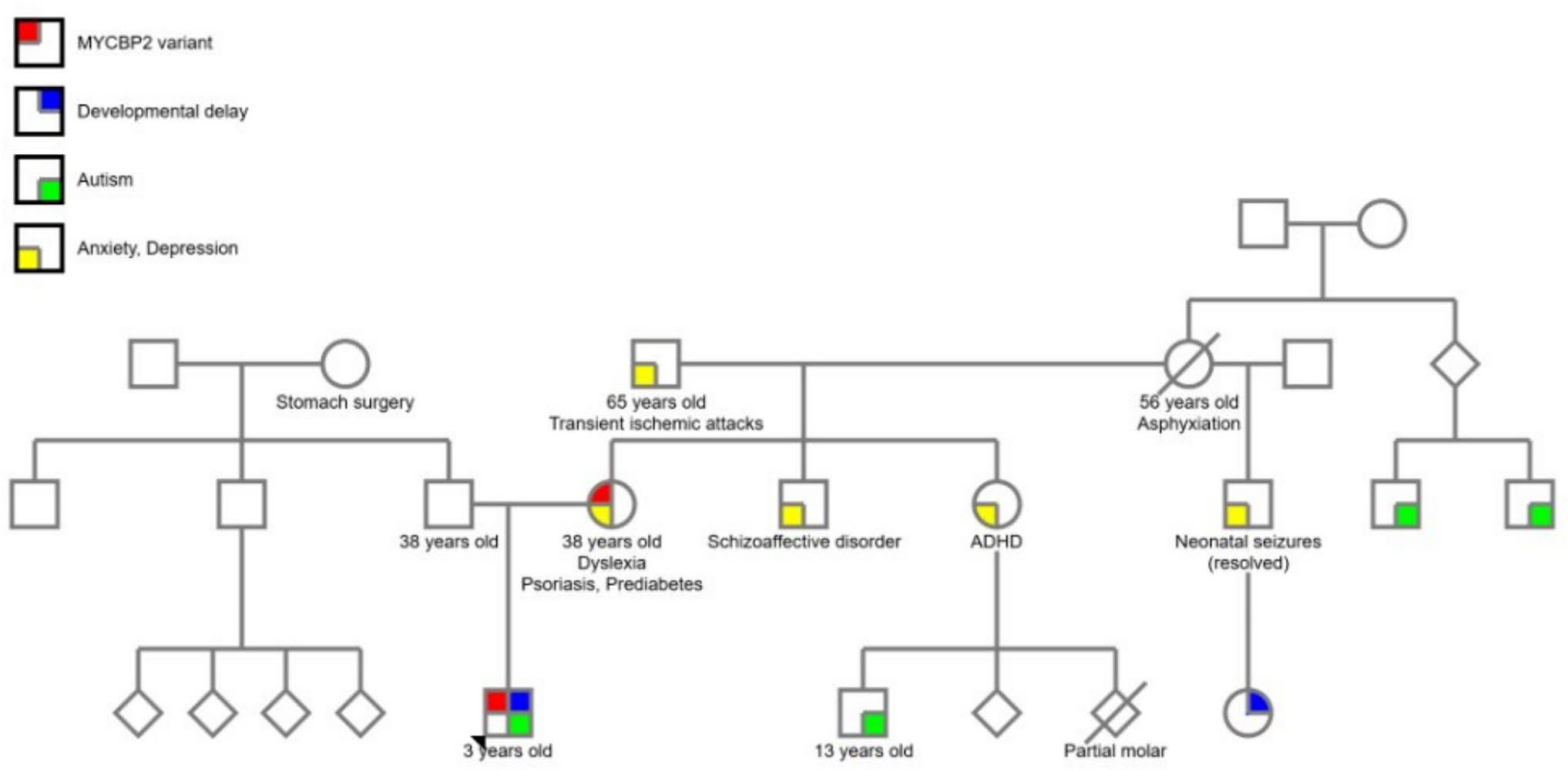
Family pedigree of proband with *MYCBP2* variant. Pedigree was constructed and drawn using (Progeny Clinical Version N/Progeny Lab Version N) (Progeny Genetics LLC, Aliso Viejo, CA, www.progenygenetics.com). Permission obtained via Rightslink with Oxford Journals for reproduction after formal license acquired

Family history is remarkable for depression, anxiety, and dyslexia in the proband’s mother. Extended family history was significant for anxiety, depression, attention-deficit/hyperactive disorder, developmental delays, and ASD. A family pedigree is shared (Fig. 1).

Subspecialty care includes gastroenterology, developmental-behavioral pediatrics, and ophthalmology. No dysmorphic facial features were noted on physical exam.

### Genetic and imaging assessments

First-line genetic testing included negative chromosomal microarray and negative Fragile X testing. Trio whole exome sequencing was significant for a maternally inherited heterozygous pathogenic variant in *MYCBP2 (NM_015057.4)*, c.4409dup (p.Leu1470Phefs*7) resulting in a diagnosis of *MYCBP2*-related neurodevelopmental disorder. For this gene, 99.6% of the coding region was covered at a minimum of 10 × with mean depth of coverage at 75 × and quality threshold of 97.8%. The lab reported 100% coverage for this area on review. There is no indication of a multi-exon deletion/duplication involving this gene in the sequencing data. The read ratio between wild-type and mutant alleles in the mother’s sample was 98% at a minimum of 10x. Mosaicism was not assessed. Upon manual re-review of the exome data by the testing lab, the *MYCBP2* c.4409dup:p.(Leu1470Phefs*7) variant was present in 47% of 55 sequencing reads in mother #2800429, it was present in 40% of 52 sequencing reads in proband #2800117. Variant was absent in father #2800441 with 0% in 81 sequencing reads.

**Fig. 2:**
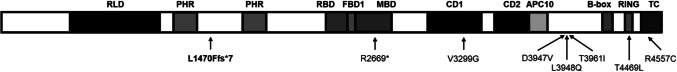
*MYCBP2* variants reported in patients with neurodevelopmental disorders. The L1470Ffs*7 variant reported in our patient yields a more truncated gene product than previously reported variants. Variant was constructed and drawn using Microsoft Office 365 PowerPoint™. Original gene visual can be found in AlAbdi et al. [1]

In contrast to the reported de novo cases [1], the variant found for this proband was substantially truncated (Fig. 2).

The proband had an MRI brain with and without contrast which showed a normal corpus callosum. There was a cluster of non-specific punctate white matter lesions in the right centrum semiovale that were likely related to his history of prematurity. The proband had a normal eye exam performed under sedation.

### Proband ASD assessment

Proband: ASD assessment was initiated at 31 months which included Diagnostic and Statistical Manual of Mental Disorders (DSM-5-TR) [2] clinical interview and standardized parent report of adaptive skills and sensory concerns obtained from mother and direct testing with the Childhood Autism Rating Scale, Second Edition, Standard Version (CARS2-ST) [3] and the Screening Tool for Autism in Toddlers and Young Children (STAT) [4] by testing clinician. Patient had limited communication including no expressive speech, vocalizations, nor use of communicative gestures at that time.

### Neuropsychological assessments

Neuropsychological testing with the proband was requested following autism testing to assess current development level and establish a baseline for monitoring development over time. Neuropsychological testing was completed at 3 years of age and was limited due to behavior and a high activity level (e.g., frequently running around the testing room or climbing on the table). He also frequently attempted to put non-food objects in his mouth. He often pulled at the examiner’s arms to attempt to take items. Comparable to the previous autism testing, the proband had limited expressive language. He was observed to say one word which was “yeah,” and he made vocalizations that included "ee," "aa," and "oo.” On tasks completed by the proband, his performance was more than two standard deviations below the average range for visual-motor integration and reasoning skills requiring visual semantic picture matching and visual-constructional skills. Parent ratings indicated that overall developmental skills including physical, social-emotional, cognitive, and communication were below age-level expectations with scores falling at or below the first percentile. Overall adaptive skills were rated by the parent as below age-level expectations, and the overall score was below the first percentile. Taken together, the evaluation results were consistent with a clinical diagnosis of global developmental delay. Scores for the testing completed with the proband as well as rating scales completed by his mother are included in Table 2.

Parent: Neuropsychological testing was also obtained for the proband’s mother and showed a largely intact profile with mild executive inefficiencies. Overall, results for testing of the proband’s mother confirm generally intact cognition with evidence of subtle executive inefficiency that did not meet criteria for a neurocognitive diagnosis, despite having a pathogenic variant in *MYCBP2*. Specifically, she demonstrated variable evidence of verbal psychomotor slowing (rapid word reading was two standard deviations below average with other scores approximately 1.5 standard deviations below average to within normal limits) and slowed fine motor dexterity (approximately two standard deviations below average). While she did not demonstrate visuospatial impairment, her approach when copying a complex figure resulted in errors/inaccuracies in line placement. Personal weaknesses were noted for animal fluency, mental arithmetic and speeded color naming (1.3 to 1.5 standard deviations below average). During the clinical interview, she denied executive functioning weaknesses in daily life with the exception of occasionally forgetting to complete tasks. Mild affective distress was also reported during the evaluation with mild anxiety symptoms endorsed on the Beck Anxiety Inventory. Clinically elevated depression symptoms were not endorsed on the Beck Depression Inventory-2. She reports a longstanding history of anxiety and depression since late adolescence. At the time of the assessment, the proband’s mother was taking Wellbutrin and Buspar as well as also participating in psychotherapy. She reported that both the medications and therapy are helpful.

Tables 1, 2 and 3 Neuropsychology tests, percentiles, and descriptors.

**Table 1:** Descriptor

Descriptor	Percentile rank
Exceptionally High	98 and above
Above Average	91 to 97
High Average	75 to 90
Average	25 to 75
Low Average	15 to 24
Below Average	3 to 14
Exceptionally Low	< 3

**Table 2:** Proband

Cognitive Domain	Scores/Percentiles	Descriptor
Tests Administered to Proband		
Reasoning		
DAS-II Picture Similarities	< 1 st percentile	Exceptionally Low
DAS-II Pattern Construction	< 1 st percentile	Exceptionally Low
Visual-Motor Integration (Beery VMI)	2nd percentile	Exceptionally Low
Receptive Language (PPVT-5)	Attempted but could not complete	
Parent-Report Measures		
Overall Development (DP-4)	< 1 st percentile	Exceptionally Low
DP-4 Subscales	Age Equivalents	
Physical	1:6–1:7	
Adaptive Behavior	1:2–1:3	
Social-Emotional	1:2–1:3	
Cognitive	1:6–1:7	
Communication	1:0–1:1	
Overall Adaptive Skills (ABAS-3)	< 1 st percentile	Exceptionally Low
ABAS-3 Subscales	Age Equivalents	
Communication	1:4–1:5	
Community Use	2:3–2:5	
Functional Pre-academics	1:0–1:1	
Home Living	1:10–1:11	
Health and Safety	1:10–1:11	
Leisure	1:6–1:7	
Self-Care	2:0–2:2	
Self-Direction	2:0–2:2	
Social	0:11	
Motor	2:0–2:2	

*DP-4* Developmental Profile 4th edition [5]; *Beery VMI* Beery-Buktenica Test of Visual Motor Integration 6th edition [6]; *PPVT-5* Peabody Picture Vocabulary Test 5th edition [7]; *DAS-II* Differential Abilities Scale 2nd edition [8]; *ABAS-3* Adaptive Behavior Assessment System 3rd edition [9]. The PPVT-5 and other portions of the DAS-II were attempted but discontinued

**Table 3:** Mother

Cognitive domain	Scores/Percentiles	Descriptor
General Intellectual Function		
WAIS-IV	FSIQ = 103	Average
Memory		
WMS-IV Logical Memory I	37th percentile	Average
Logical Memory II	37th percentile	Average
CVLT-3 Learning	16th percentile	Low Average
Long Delay	38th percentile	Average
BVMT-R Learning	76th percentile	High Average
Delayed Recall	34th percentile	Average
Attention/Executive Function		
WAIS-IV Digit Span	84th percentile	High Average
Trail Making B	21 st percentile	Low Average
Stroop Word Reading	2nd percentile	Exceptionally Low
Color Naming	10th percentile	Below Average
Interference	92nd percentile	Above Average
WCST	> 16th percentile	Within Normal Limits
Letter Fluency (COWA)	18th percentile	Average
Language		
Boston Naming Test	58th percentile	Average
Animal Fluency (COWA)	10th percentile	Below Average
Visuospatial		
RCFT Copy trial	Disorganized approach	Below Average
WAIS-IV Block Design	75th percentile	
Motor		
Grooved Pegboard—RH	3rd percentile	Below Average
Grooved Pegboard—LH	2nd percentile	Exceptionally Low

*BVMT-R* Brief Visuospatial Memory Test, Revised [10]; *CVLT-3* California Verbal Learning Test, 3rd edition [11]; *Stroop* Stroop Interference Test (Golden version [12]; *COWA* Controlled Oral Word Association Test [13]; *WCST* Wisconsin Card Sorting Test [14]; Boston Naming Test [15]; Grooved Pegboard Test [16]; *RCFT* Rey Complex Figure Test [17]; Trail Making Test [18]; *WAIS-IV* Wechsler Adult Intelligence Scale, 4th edition [19]; *WMS-IV* Wechsler Memory Scale, 4th edition [20]

### Treatments

Developmental: The proband received speech and occupational therapies in the outpatient setting from 2 to 3.5 years of age. School services with an individualized education program (IEP) under the primary eligibility of autism began at age 3 years. In the school setting, he receives specially designed instruction in cognitive/pre-academic skills (30 min, 5 days per week), communication skills (30 min, 5 days per week), adaptive behavior (45 min, 5 days per week), and social/emotional skills (30 min, 5 days per week) as well as speech therapy services (30 min, 4 per month). The proband is currently on the waitlist for applied behavior analysis therapy in the outpatient setting.

Medical: At initial evaluation, he had nighttime awakenings, snoring, restlessness during sleep initiation, and apparent night terrors. Initial intervention included primarily behavioral interventions with sleep hygiene routine. Resolution of initial sleep initiation differences and snoring as well as diminishing night terrors occurred spontaneously by follow up. Nighttime awakenings with coming into mother's bed at night leading to sleep reinitiation within 20 min of laying back down remained but were less challenging at that time.

Gastroenterology prescribed polyethylene glycol daily for constipation, starting at age 11 months. He was seen by outpatient gastroenterology at 44 months due to ongoing constipation where diet included lactose free milk, Pediasure, and some dairy, but limited vegetables or other fiber containing foods. Dietary recommendations were made including decreasing milk consumption and increasing fruits, vegetables, and water, screening lab work and an x-ray of the abdomen were ordered. He was subsequently admitted for inpatient gastroenterology cleanout for fecal impaction, after which his regimen consisted of polyethylene glycol daily and sennoside every other day.

Developmental-behavioral pediatrics started guanfacine for aggressive behavior at age 3 years.

## Discussion

In the initial study describing *MYCBP2*-associated neurodevelopmental disorder, all individuals had de novo heterozygous pathogenic variants. We describe here the first case of an inherited *MYCBP2*-associated neurodevelopmental disorder in a pediatric patient inherited from a less-affected parent, expanding the phenotype of this neurodevelopmental disorder to include isolated mild executive dysfunction. The proband’s neurobehavioral phenotype was consistent with previous description, including significant developmental delay and diagnosis of ASD, but he did not have the previously described ocular or corpus callosum findings. Development of epilepsy/seizures has not been seen, but the proband is being monitored. From a cognitive perspective, it is possible that a diagnosis of intellectual developmental disorder may be warranted.

Proband and parent both received evaluation with neuropsychology, which makes this the first published neuropsychological profile in a 2-generation sample and demonstrates the presence of subtle neurocognitive changes compared to same-aged peers in a mildly affected parent. These skill differences may impact both the parent’s daily functioning across work and home as well as their ability to attain care and access interventions for their children with neurodevelopmental disorders [21, 22]. Therefore, identification of a clinically significant variant in a parent should prompt evaluation even if no concerns or issues were previously self-reported. In this dyad, mother is currently deciding how to share this new information within the family at large. Currently any impact for other family members is not yet known, but future testing for maternal relatives could yield important information about both inheritance and variable expressivity in the family as a future addition to the understanding of this variant.

Neuropsychological assessment and the selection of appropriate measures to track changes related to neurocognitive function over time is an important component of understanding natural disease course [23, 24]; although, there are no clear, specific guidelines for neuropsychological assessment in children with rare genetic diagnoses due to several factors. The proband’s behavior made it challenging to obtain estimates of his cognitive skills, and as such, caregiver rating scales were used to assist with better understanding his development. During these evaluations, it is important to consider limitations and benefits of assessment measures (e.g., cultural sensitivity; lowest level of performance reliably detected). The process for assessment of family members incidentally found to have pathogenic variants in genes associated with neurodevelopmental disorders is also undefined, but our expanding understanding of *FMR1*-related disorders in patients with > 200 CGG repeats compared to family members with indeterminate or intermediate repeat expansion highlight the importance of delineating the variable phenotypic expression of symptoms within families [25]. Additionally, the emergence of age-related phenotypes within this same disorder demonstrate the importance of iterative longitudinal assessments of affected individuals at every stage of life [26]. Prior literature reports that assessment of full parental genotype and phenotype can help to anticipate a child’s developmental trajectory and future needs, especially in genetic disorders associated with highly variable expressivity [27]. Although we recognize that families value information about neurodevelopmental outcomes that provide practical information for their child [28] a significant gap in our understanding of the familial impact of inherited neurodevelopmental disorders across generations remains.

## Data Availability

No datasets were generated or analysed during the current study.
